# Bacteremia caused by *Helicobacter cinaedi*: a case report

**DOI:** 10.1515/almed-2021-0092

**Published:** 2021-12-30

**Authors:** Kateryna Sidak, Ramón Pérez-Tanoira, Peña Gomez-Herruz

**Affiliations:** Service of Laboratory Analysis, Príncipe de Asturias University Hospital, Alcalá de Henares, Madrid, Spain; Service of Microbiology, Príncipe de Asturias University Hospital, Alcalá de Henares, Madrid, Spain; Department of de Biomedicine and Biotechnology, School of Medicine, University of Alcalá, Alcalá de Henares, Spain

**Keywords:** bacteremia, *Helicobacter cinaedi*, Maldi-TOF

## Abstract

**Objectives:**

*Helicobacter cinaedi* is a Gram-negative, spiral-shaped bacterium that primarily affects immunosuppressed patients.

**Case presentation:**

A 49-year-old patient with ulcerative colitis diagnosed in 1992, who presented to the ED of our hospital with fever and testicular complaints. The patient was discharged with a diagnosis of left-sided acute epididymitis, which was probably sexually transmitted. At the ED, he was administered intravenous Ceftriaxone and discharged with a prescription of doxycycline for 10 days, with a good progress. Aerobic cultures were positive at three days from collection. Gram staining showed Gram-negative, corkscrew-shaped bacteria. The analysis of the blood culture bottles, and the colonies grown in Campylosel agar incubated in microaerophilic conditions at 42 °C were identified as *H. cinaedi* on the Maldi-TOF Biotyper 3.0 system (Bruker Diagnostics Inc.).

**Conclusions:**

Direct analysis of the blood culture bottle on the Maldi-TOF system allowed for the identification of the etiology of the bacteremia since *H. cinaedi* could not have been grown in standard culture conditions. The treatment of this infection is a matter of debate; however, the combination of ceftriaxone with doxycycline can be ineffective for bacteremia caused by *H. cinaedi* infection since it occurs by the translocation of the bacteria from the gastrointestinal tract. This type of bacteremia is associated with intestinal mucosal damage secondary to ulcerative colitis, and it primarily affects immunosuppressed patients.

## Introduction

*Helicobacter cinaedi* is a Gram-negative, spiral-shaped bacterium. This bacterium was first isolated in 1984 by Fennel in the rectal specimens of homosexual men with proctocolitis [1]. *H. cinaedi* may cause gastroenteritis, cellulitis, meningitis, rash, or bacteremia and mostly affects immunosuppressed patients, although it may also affect immunocompetent patients [2, 3]. Hamsters are the natural reservoir, though it has been isolated in fecal material of dogs and cats [4].

## Case presentation

These bacteria are rarely found in our environment. For this reason, we report a case of bacteremia caused by *H. cinaedi* in a 49-year-old man receiving treatment with infliximab, who presented to our hospital with fever and testicular complaints. The patient reported having had unprotected sex. On physical examination, left testicular enlargement was noted, with the tail of the epididymis being thickened and painful. No urethral secretion was observed. Laboratory test excluded leukocytosis, but C-reactive protein was elevated. Urine showed pyuria and moderate hematuria. Urine culture demonstrated >100,000 colony forming units/ml of *Escherichia coli*. The patient was diagnosed with ulcerative colitis in 1992 and had been hospitalized for this cause two times. The first time, he was treated with cyclosporine, the second with mercaptopurine. The patient was hospitalized in 1997 for a new flare up of corticosteroid-resistant ulcerative colitis. Fecal analysis revealed the presence of *Campylobacter jejuni*. In 2019, he had acute right-sided epididymitis and was diagnosed with syphilis and gonorrhea. This time, the patient was discharged with a diagnosis of acute, left-sided epididymitis, which had been probably sexually-transmitted, and was treated with doxycycline 100 mg twice daily for 10 days. Prior to discharge, a dose of 250 mg of ceftriaxone was administered intravenously, and two paired blood cultures were incubated in Biomerieux Virtuo BacT/ALERT for three days, until overgrowths were detected in the aerobic bottles of the two cultures. Overgrowths were not detected in the two anaerobic bottles.

According to Araoka et al. [5], automated systems take a mean of five days to detect the growth of *H. cinaedi* in blood-culture bottles (2–12 days). Gram-stained smear of the blood-culture medium revealed Gram-negative, corkscrew-shaped bacteria. Direct analysis of the content of the two aerobic blood-culture bottles was performed on a Maldi-TOF Biotyper 3.0 system (Bruker Diagnostics Inc.) Analysis demonstrated the presence of *H. cinaedi* with a score of 1.7. Blood cultures showed no overgrowth on the chocolate agar plates (Biomerieux) incubated at an atmosphere of 5% of CO_2_. In contrast, overgrowths were observed on the Campylosel agal (Biomerieux) plate, which is selective for *Campylobacter,* incubated in microaerophilic conditions on a BD GasPack EZ Pouch System at 42 °C. *H. cinaedi* grows at 35 °C, although some strains have been reported to grow at 42 °C [5, 6]. The colonies grown on Campylosel plates on Maldi-TOF Biotyper 3.0 confirmed the presence of the previously identified *H. cinaedi*. The antimicrobial sensitivity of this microorganism could not be assessed by the Kirby-Bauer method because there was no overgrowth of the bacterium isolated on Campylosel agar in microaerophilic conditions.

When blood culture results were delivered to the patient, symptoms had already disappeared after antibiotic treatment with doxycycline was completed at home.

## Discussion

The etiology of bacteremia could be established by the identification of Gram-negative, spiral, corkscrew-shaped bacteria on the positive Gram-stained smear of blood culture medium and direct analysis of the medium by the Maldi-TOF method. This would not have been possible with a chocolate agar medium subculture from positive blood bottles.

Since most of the evidence available is for *Helicobacter pylori*, the optimal treatment for this infection is a matter of debate. In general, *H. cinaedi* is sensitive to carbapenems, aminoglycosides, tetracyclines, penicillins, and cephalosporins, and it is resistant to macrolides. Resistance to quinolones secondary to a DNA gyrase mutation has also been reported [1]. Different treatments for this type of bacteriemia have been proposed in the literature: Bateman et al. [7] used a combination of ceftriaxone and doxycycline for two weeks in a patient with concurrent multiple myeloma. Lacruz et al. [2] administered imipenem and gentamicin for two weeks and doxycycline for five weeks to a patient with hepatitis C. Suzuki et al. [3] treated a patient with a hepatic cyst with ampicillin/sulbactam*.*

Although it is rare, *H. cinaedi* can colonize the gastrointestinal tract, and translocation may result in the development of bacteremia associated with the intestinal mucosal damage caused with ulcerative colitis [5]. This primarily occurs in immunosuppressed patients.

## Learning points

– In most cases, *H. cinaedi* affects immunosuppressed patients.
– *H. cinaedi* is a highly-demanding, slow-growing microorganism. In the case presented here, isolation was performed on Campylosel agar in microaerophilic conditions incubated at 42 °C, which delays diagnosis.
– Blood culture Gram staining is crucial for the detection of the presence of *H. cinaedi.* However, its characteristics may be misleading, and it may be mistaken for *Campylobacter* spp.
– The isolation and identification of *H. cinaedi* is challenging, and Maldi-TOF mass spectrometry is extremely useful.
– Although a standard treatment has not yet been established, there is evidence of effective treatments in the literature.
– *H. cinaedi* is an enterohepatic species of *Helicobacter* and a rare cause of gastroenteritis and bacteremia.
